# Genetically-Based Olfactory Signatures Persist Despite Dietary Variation

**DOI:** 10.1371/journal.pone.0003591

**Published:** 2008-10-31

**Authors:** Jae Kwak, Alan Willse, Koichi Matsumura, Maryanne Curran Opiekun, Weiguang Yi, George Preti, Kunio Yamazaki, Gary K. Beauchamp

**Affiliations:** 1 Monell Chemical Senses Center, Philadelphia, Pennsylvania, United States of America; 2 Battelle - Pacific Northwest Division, Richland, Washington, United States of America; 3 Department of Dermatology, School of Medicine, University of Pennsylvania, Philadelphia, Pennsylvania, United States of America; Duke Unviersity, United States of America

## Abstract

Individual mice have a unique odor, or odortype, that facilitates individual recognition. Odortypes, like other phenotypes, can be influenced by genetic and environmental variation. The genetic influence derives in part from genes of the major histocompatibility complex (MHC). A major environmental influence is diet, which could obscure the genetic contribution to odortype. Because odortype stability is a prerequisite for individual recognition under normal behavioral conditions, we investigated whether MHC-determined urinary odortypes of inbred mice can be identified in the face of large diet-induced variation. Mice trained to discriminate urines from panels of mice that differed both in diet and MHC type found the diet odor more salient in generalization trials. Nevertheless, when mice were trained to discriminate mice with only MHC differences (but on the same diet), they recognized the MHC difference when tested with urines from mice on a different diet. This indicates that MHC odor profiles remain despite large dietary variation. Chemical analyses of urinary volatile organic compounds (VOCs) extracted by solid phase microextraction (SPME) and analyzed by gas chromatography/mass spectrometry (GC/MS) are consistent with this inference. Although diet influenced VOC variation more than MHC, with algorithmic training (supervised classification) MHC types could be accurately discriminated across different diets. Thus, although there are clear diet effects on urinary volatile profiles, they do not obscure MHC effects.

## Introduction

For social animals, an ability to identify individuals is almost a prerequisite for the efficient organization of behavioral interactions. Many studies have demonstrated that individual animals can recognize one another as individuals and that, for mammals in particular, body volatiles (herein referred to loosely as odors) detected by olfactory or vomeronasal receptors play a prominent role in mediating this individual recognition [1]. We have previously proposed that each individual mouse has a unique odor which we have termed its odortype [2]. Odortypes, like other phenotypes, are influenced by genetic and environmental variation, and possibly their interaction. Among the genetic bases for individual odortypes, variation in genes of the MHC plays a central role as we and many others have demonstrated [2]–[5]. Variation in non-MHC genes can also influence the urinary odors of mice and contribute toward specification of genetically-determined odortypes [6]–[8].

Individual identity, as it is commonly conceived, is the sum of the characteristics of an individual animal that distinguish it from other members of its species. It is generally assumed that these individual characteristics must be relatively stable over considerable time periods so that the individual can be recognized in multiple behavioral and social contexts. Thus for odortypes it would seem desirable that they be stable over time and relatively uninfluenced by day-to-day fluctuations due to such factors as variation in diet.

Nevertheless, there is considerable evidence suggesting short-term fluctuations in body odors due to variation in stress [9], disease state [10], [11] and diet [12], [13 and reference therein]. Some reports suggest that dietary changes might mask genetically determined odortypes, preventing individual recognition [14], [15]. However, it would be surprising if an altered diet made it impossible to recognize genetically-determined individuality of odor, as Schellinck et al [15] suggest. Instead, we hypothesize that genetically-determined odortypes, and particularly MHC-determined odortypes, are relatively buffered against changes due to short-term environmental fluctuations. Such buffering would seem to be a prerequisite for MHC odortypes to be involved in mediating mate choice in natural environments, as studies in semi-natural testing conditions suggest [16].

To clarify the influence of diet on MHC-regulated odortypes, we conducted combined behavioral and chemical studies using urine samples from two different congenic mouse strains each on two different diets. First, we tested whether MHC odortypes are perceived following substantial changes in diet. We found that although diet clearly has a large effect on urinary odors, MHC-determined odortype variation can be recognized in spite of major diet variation. Chemical analyses of urinary VOCs for these same mice were completely consistent with behavioral results: dietary variation significantly altered the profile of urinary VOCs, but a clear subset of MHC determined VOCs was unperturbed by diet variation, allowing for statistical discrimination of MHC types across dietary treatments.

## Results

### Behavior

#### Experiment 1

Results for mice reinforced for alternative choices were not significantly different, so the data are shown combined, as well as separately, in Table 1. The combined concordance (that is correct response) score for the generalization trials of coded urine samples where only MHC varied was 55% (not different from chance) whereas when only diet varied it was 75% (significantly different from chance), suggesting that mice trained to discriminate urines from mice that differed both in diet and MHC type found the diet odor more salient. Because sensor mice did not respond to MHC differences in generalization trials (in which diet was held constant), one might conclude that diet obscures MHC odortypes. But such a conclusion is premature. The second experiment approached this question in a slightly different way.

**Table 1:** Responses to urine odor choices for Behavior Experiment 1.

Sensor mouse	Different MHC, Same Diet*				Different Diet, Same MHC			
	Both on Diet S		Both on Diet L					
	B6 vs B6-H2^k^		B6 vs B6-H2^k^		B6 vs B6		B6-H2^k^ vs B6-H2^k^	
	**+ ** a	**−**	**+**	**−**	**+**	**−**	**+**	**−**
1† B6♀	11^b^	9	9	9	15	3	15	4
2† B6-H2^k^♀	13	6	9	7	14	4	14	4
3‡ B6♀	10	8	9	3	13	5	8	6
4‡ B6♀	10	9	9	9	14	4	15	2
5‡ B6-H2^k^♀	6	13	12	7	14	4	11	8
**Totals**	**50**	**45**	**48**	**35**	**70** §	**20**	**63** §	**24**
	**55% (98/178)**				**75% (133/177)**			

Mice trained to discriminate urine odors of mice differing in both the MHC and in diet generalized this response to diet changes but not to MHC.

* Diet: Diet S = Synthetic diet; Diet L = Laboratory Rodent diet.

† on training trials reinforced to go to B6 Diet S over B6-H2^k^ Diet L.

‡ on training trials reinforced to go to B6-H2^k^ Diet S over B6 Diet L.

§ p<0.0001, binominal test.

a The plus sign (+) refers to a choice that is correct or concordant with training whereas the minus sign (−) refers to a response that was incorrect or not concordant with training.

b The numbers in the tables represent the generalization trials only.

#### Experiment 2

As in Experiment 1, results for alternative training modes were not significantly different, so the data are shown combined, as well as separately, in Table 2. A combined concordance score of 90% (p<0.0001, binomial test) was attained in training trials on laboratory diet (termed Diet L). The combined concordance score for the generalization trials of coded urine samples from mice fed on synthetic diet (Diet S) not previously encountered was also 90% (p<0.0001, binomial test), signifying that some components of the MHC-determined pattern of volatiles are preserved in spite of variation induced by the dietary change.

**Table 2:** Responses to urine odor choices for Behavior Experiment 2.

Sensor mouse	Training trials: Diet L*		Generalization trials: Diet S†	
	**+** a	**− **	**+**	**−**
6 B6♂	43	3	9	0
7 B6♂	35	5	6	1
8 B6♂	49	3	10	0
9 B6-H2^k^♀	51	13	8	1
10 B6-H2^k^♀	40	2	8	0
11 B6♂	41	2	6	2
12 B6♂	39	3	6	2
13 B6♂	49	5	10	0
14 B6♀	44	3	7	2
15 B6-H2^k^♀	42	12	8	1
16 B6-H2^k^♀	41	3	8	1
**Totals**	**474** ‡	**54**	**86** ‡	**10**
	**90%**		**90%**	

Mice trained to discriminate MHC differences on Diet L generalized this response to the same differences in MHC genes on the different diet (Diet S).

* Diet L = Laboratory Rodent diet.

† Diet S = Synthetic diet.

‡ p<0.0001, binominal test.

a The plus sign (+) refers to a choice that is correct or concordant with training whereas the minus sign (−) refers to a response that was incorrect or not concordant with training.

In combination, Experiments 1 and 2 suggest that when compared directly, diet variation can be more salient to trained mice than MHC variation in regulating urinary odors. However, in spite of this, the MHC-determined odortype can be recognized against variation induced by dietary changes.

### Chemistry

**Figure 1:**
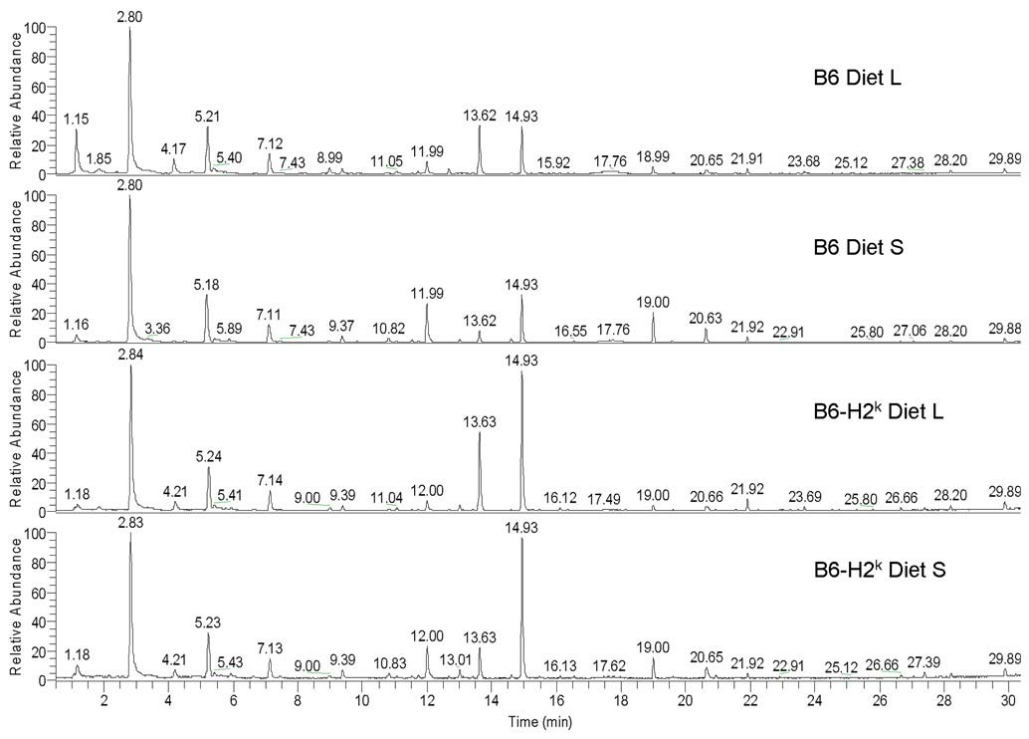
Typical total ion chromatograms from the urinary volatiles extracted from four mouse groups. One representative chromatogram each from B6 mice fed Diet L, B6 mice fed Diet S, B6-H2^K^ mice fed Diet L and B6-H2^K^ mice fed Diet S is shown here. Prominent compound peaks include 2-acetyl-1-pyrroline (11.99 min), beta-farnesene (19.00 min), alpha-farnesene (20.63 min), and the mouse pheromones 2,3-dehydro-exo-brevicomin (13.62 min) and 2-sec-butyl-4,5-dihydrothiazole (14.93 min).

Figure 1 shows four typical total ion chromatograms (TICs) from analyses of urinary VOCs extracted by SPME from the four mouse groups (C57BL/6J (B6) Diet L, B6 Diet S, C57BL/6J-H2^k^ (B6-H2^k^) Diet L, and B6-H2^k^ Diet S). Statistical analysis of the GC/MS data collected from the 37 individual mice was performed to identify differentially-expressed compounds, many of which might co-elute with other peaks. Over 100 distinct chromatographic components were detected and we were able to identify 25 of them and another 24 compounds were tentatively identified, as seen in Table 3. Separate analyses were performed using the full profile of chromatographic components and the 49 identified components, with the same general conclusions. Unless otherwise stated, reported results are based on analysis of the 49 identified compounds so that conclusions could be attributed to known compounds, and to remove any concern that reported differences are due to instrumental artifacts or contamination. All compounds were present in each of the four mouse groups, suggesting that the differences in urinary VOCs between four groups are determined by the relative proportions of the compounds rather than by the presence or absence of certain compounds. Several of the compounds listed in Table 3 appear to be of exogenous origin (diet or environment). For example, 4-heptanone, 2-ethylhexanol and 2-ethylhexanoic acid are derived from plasticizers and their metabolites [17] and 1-methyl-4-(1,2,2-trimethylcyclopentyl)-benzene and 1-(1,1-dimethylethyl)-2-methyl-1,3-propanediyl 2-methylpropanoate are similar to volatile constituents from painted wallboard [18]. Several terpenes (e.g. thujopsene) are detected in mouse bedding materials (unpublished data) or may be their metabolites.

**Table 3:** Compounds identified in mouse urine, and their statistical significance.

id#	compound	diet	MHC	interaction
1	2,3-pentanedione	0	0.001	0.045
2	dimethyl disulfide	0.79	0.231	0.901
3	***4-heptanone***	0	0	0.102
4	3-penten-2-one	0.443	0	0.184
5	nitromethane	0.071	0.003	0.155
6	***beta-myrcene***	0.794	0.071	0.334
7	1,4-cyclohexanedione (T)	0.453	0.209	0.926
8	2-heptanone	0.369	0.001	0
9	6-methyl-3-heptanone (T)	0.293	0.172	0.001
10	3-methylcyclopentanone	0.023	0	0.003
11	2-pentenyl acetate (T)	0.297	0	0.152
12	5-hepten-2-one (T)	0	0	0.009
13	3-hydroxy-2-butanone	0.399	0.034	0.15
14	1-hydroxy-2-propanone	0.415	0.612	0.853
15	4-methyl-6-hepten-3-one (T)	0.012	0.137	0.001
16	3-ethylcyclopentanone	0.001	0.146	0.696
17	2-acetyl-1-pyrroline (T)	0.075	0	0.503
18	2-isopropyl-4,5-dihydrothiazole (T)	0.303	0.004	0.001
19	2-sec-butylthiazole	0.063	0.438	0.391
20	2,3-dehydro-exo-brevicomin (T)	0	0.514	0.754
21	1-octen-3-ol	0	0.055	0.721
22	4-hydroxy-2-pentanone (T)	0	0.064	0.755
23	2-sec-butyl-4,5-dihydrothiazole (T)	0.006	0.042	0.034
24	***2-ethylhexanol***	0.266	0.361	0.958
25	***1-(2-methoxy-1-propoxy)-2-propanol (T)***	0.571	0.21	0.411
26	a terpene (T)	0	0.044	0.757
27	benzaldehyde	0.087	0.019	0.473
28	***linalool***	0	0.617	0.917
29	3,6-heptanedione (T)	0.016	0.002	0.765
30	unknown	0.502	0.018	0.117
31	***thujopsene (T)***	0.013	0.865	0.936
32	beta-farnesene (T)	0.422	0.168	0.574
33	methyl methylthiomethyl disulfide (T)	0.03	0.001	0.32
34	isovaleric and 2-methylbutyric acids	0	0.293	0.946
35	γ-caprolactone	0.046	0.354	0.139
36	benzyl methyl ketone (T)	0.084	0.929	0.745
37	alpha-farnesene (T)	0.415	0.18	0.55
38	o-toluidine (T)	0	0.619	0.238
39	***1-methyl-4-(1,2,2-trimethylcyclopentyl)-benzene (T)***	0.05	0.02	0.613
40	***1-(1,1-dimethylethyl)-2-methyl-1,3-propanediyl 2-methylpropanoate (T)***	0.097	0.007	0.102
41	dimethyl sulfone	0	0	0.009
42	dodecanol	0.16	0.18	0.705
43	***2-ethylhexanoic acid***	0.807	0.108	0.073
44	2-acetyl pyrrole	0.076	0.001	0.666
45	***levoglucosenone (T)***	0	0.003	0.467
46	phenol	0.255	0	0.021
47	***nerolidol (T)***	0.538	0.039	0.537
48	p-cresol	0.883	0	0.837
49	p-ethyl phenol	0	0	0

The compounds are listed in the order of increasing retention time. The numbers in the right-hand columns of Table 3 are the p values and a zero means p≤0.0001. (T) = tentatively identified. Compounds likely to be of exogenous origin are printed in ***bold-italic***-face type.

Model [1] (see Materials and Methods, Data analysis) was fit separately for each of the 49 compounds. Compounds and their significance levels for three separate effects (Diet, MHC, and MHC×Diet) are shown in Table 3. The numbers in the right-hand columns of Table 3 are the p values and a zero means p≤0.0001. For example, 2,3-dehydro-exo-brevicomin (id# 20) is affected by diet (p≤0.0001). Using the false discovery rate procedure of Benjamini and Hochberg [19] to adjust p-values for multiple statistical tests, at *α* = .1 we find 20 compounds influenced by Diet, 20 compounds influenced by MHC, and 10 compounds for which there is a significant Diet×MHC interaction. Of the 10 compounds with significant interaction effects, 8 are crossover interactions.

**Figure 2:**
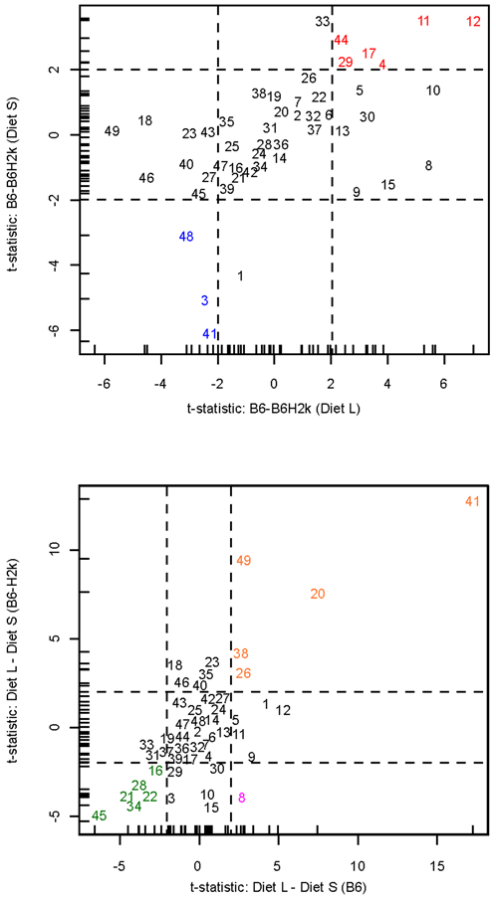
Comparison of t-statistics for within-diet MHC effects compared for two diets (top), and within-MHC Diet effects compared for two MHC types (bottom). Each number represents a compound, indexed in Table 3. Two separate test statistics are represented on horizontal and vertical axes. Horizontal and vertical dashed lines represent thresholds for statistical significance, so that the middle of central panel represents non-significance for both tests. Regarding the relative concentration of a certain compound, in the top panel, red color represents compounds where the concentration is higher in B6 than in B6-H2^k^ and blue color represents compounds where the concentration is higher in B6-H2^k^ than in B6 regardless of diet. In the bottom panel, orange color represents compounds where the concentration is higher in Diet L than in Diet S and green color represents compounds where the concentration is higher in Diet S than in Diet L regardless of MHC type. The pink color represents the single compound where the concentration is higher in Diet L under B6 MHC type, but is lower in Diet L under B6-H2^k^ MHC type.

To further understand the influence of MHC×Diet interactions, and the stability of MHC effects across dietary treatments, we plotted t-statistics for MHC differences for a single dietary treatment verses the corresponding t-statistic for MHC differences on a different dietary treatment (see top panel of Figure 2). The positive correlation in the t-statistics of MHC differences across the two different diets (horizontal axis versus vertical axis) is an indication of MHC stability (or the relatively small influence of interactions). The bottom panel of Figure 2 compares t-statistics for diet differences, computed separately for each MHC type. Horizontal and vertical dashed lines represent thresholds for statistical significance, so that the middle of central panel represents non-significance for both tests. The threshold lines correspond to an approximate unadjusted p-value of .05 and false discovery rate of .10. Regarding the relative concentration of a certain compound, in the top panel, a positive threshold value indicates that the concentration is higher in B6 than in B6-H2^k^, whereas a negative one indicates the concentration is higher in B6-H2^k^. Likewise, in the bottom panel, a positive value indicates that the concentration is higher in Diet L than in Diet S, while a negative one indicates the concentration is higher in Diet S. In the top panel, for example, the threshold values of 2,3-dehydro-exo-brevicomin (id# 20) fall near zero on both axes, indicating no significant MHC effect. In the bottom panel, however, the values are away from zero (positive) on both axes which are an indication of a significant diet effect. Its concentration is higher in Diet L than in Diet S regardless of MHC type. In the case of 3-penten-2-one (id# 4), the positive values on both axes of the top panel indicate that its concentration is higher in B6 mice than in B6-H2^k^ mice regardless of diet. The values for this compound in the bottom panel fall near zero on both axes, indicating no significant diet effect.

Multivariate statistical methods were used to further characterize the effects of MHC and Diet on VOCs. Redundancy analysis was used to estimate the relative contributions of Diet, MHC, and MHC×Diet to the total non-redundant systematic variability. Using this approach, 53% of total variability was attributed to Diet, 35% to MHC, and 12% to their interaction.

Finally, we conducted a Random Forest decision tree analysis using the R package *randomForest*, which perturbs or bootstraps data many times (10,000 in this case) and constructs a separate tree for each perturbation. Suspected exogenous compounds were excluded from this analysis (see Table 3). In 76% of trials the first split primarily divided samples based on diet; in 24% the division was on MHC, suggesting that diet has a greater influence than MHC on VOC variation. An unbiased estimate (“out of bag”, based on samples not used to fit the tree) of error rate for classifying samples to one of four groups is 16%.

**Figure 3:**
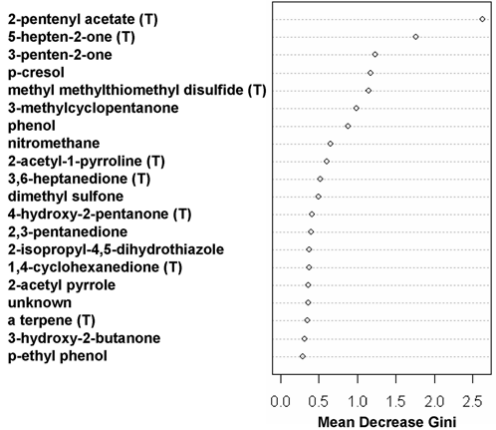
Endogenous compound ranked by their importance in discriminating MHC types across diets. The importance measure is assessed by mean decrease in the Gini index (higher values are more important), a relative measure of group (MHC) differences explained. See the text for details. (T) = tentatively identified from mass spectral data.

**Figure 4:**
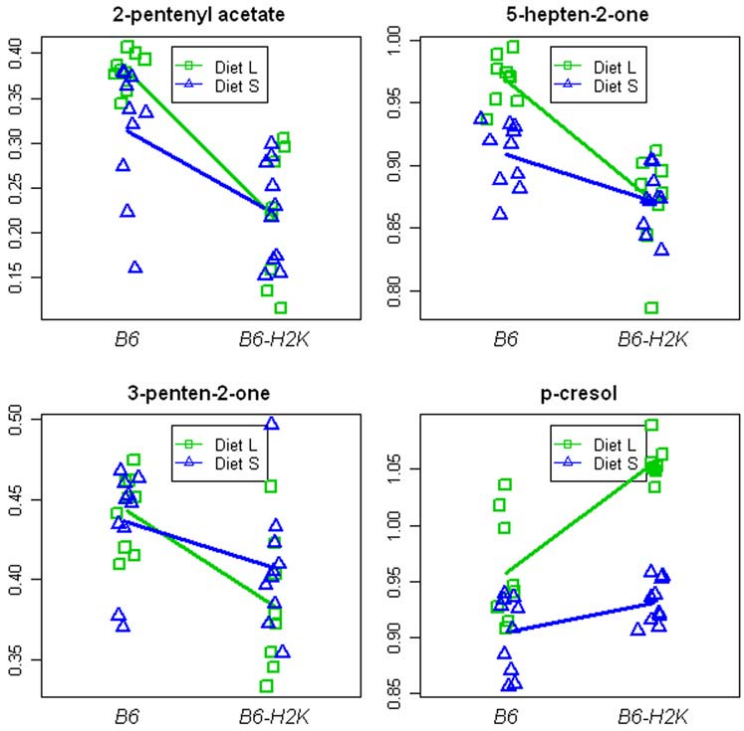
Distributions of the intensities for the top-ranked compounds affected by MHC types. Each panel displays normalized intensity values (y-axis) for a single metabolite affected by MHC types. MHC type is given on the x-axis, and different diets are distinguished by color and plotting character (green square for Diet L; blue triangle for Diet S). A line connecting average responses for each MHC type is shown separately for Diet L (green) and Diet S (blue).

The same Random Forest method was used to assess how well MHC types could be discriminated amid varying diets. To mimic the sensor mouse discrimination, we constructed a Random Forest classifier to discriminate MHC types for mice on normal diet, and applied the classification rule to mice on synthetic diet. Then we constructed a Random Forest classifier to discriminate MHC types for mice on synthetic diet, and applied the classification rule to mice on normal diet. Across both test sets (containing 37 samples), 6 errors were made, resulting in an error rate of 16%, significantly better than chance. (Using the entire components we get 7 errors.) This error rate translates into an 84% correct classification, close to the 90% found in mouse behavioral testing (Table 2). Compounds that contribute to this prediction are ranked in Figure 3 according to their relative importance and the distributions of the normalized intensities for the top-ranked compounds affected by MHC types are illustrated in Figure 4. Variability between individual observations is greater than we have seen in previous studies [6], [20], probably because lower concentration of urine was used.

## Discussion

A major difference in diet has more effect on the total urinary odor profile – as measured behaviorally with trained mice or chemically – than do MHC differences. Nevertheless, MHC difference can be recognized even when confounded by major dietary effects (see Figures 2, 3 and 4). The interaction between diet and MHC type explained roughly 12% of VOC variation. This represents a smaller interaction effect than we had found between MHC type and background genetic variation (∼25%; 6). It is noteworthy that in both cases, trained mice were able to recognize the MHC variation in spite of these interactions. This implies that there is a specific subset of compounds the relative concentrations of which reflect MHC type but which are not influenced by genetic or environmental variation. Other behavioral data are consistent with this hypothesis [16], [21].

Some compounds that are regulated by MHC have been tentatively identified [20], [22]. For example, 2-sec-butyl-4,5-dihydrothiazole is higher in B6-H2^K^ urines compared to B6 urines, as was found with previous urine analyses that employed a solvent extraction method [20] and the same SPME method [6]. Dimethyl sulfone, which was also reported to be an MHC-regulated compound [20], is affected additively by both diet and MHC. However, a number of the previously reported compounds were not detected in this study or did not significantly differ between the congenic mice. Some of these differences can be attributed to differences in the methods used to collect and isolate the samples. In earlier studies, bioassays implicated the acidic fraction of solvent-extracted urine as carrying the olfactory-active compounds of the signal [23], [24]. This extraction method involved centrifugal ultrafiltration to remove MUPs (known to be involved in mouse behavioral regulation [25], [26]), ion exchange chromatography, lyophilization, pH adjustment, and solvent extraction. Since many steps are involved, some compounds were surely lost or diminished during the extraction. For example, several mouse pheromones are bound to MUPs [27] and these compounds may be partially or completely lost during the centrifugal ultrafiltration step. In addition, highly volatile compounds are easily lost due to the long extraction procedure.

More recently we have collected volatiles using the SPME method as described here. SPME, invented by Pawliszyn [28] is a simple and efficient collection method for VOCs. It has been widely used in different fields of analytical chemistry. This technique has been employed for the detection of a variety of mouse urinary VOCs including several putative pheromones [29], [30]. Volatiles collected by this method carry sufficient information for trained mice to identify MHC type [31]. However, this SPME method generally does not detect phenyl acetic acid, a previously reported MHC-regulated compound [24] and other less volatile compounds, unless a large volume of urine is extracted. Therefore, the relative concentrations and profiles of the urinary VOCs vary, depending on which extraction method is used, and additional work combining several methods is needed to provide a complete list of compounds that vary according to MHC type.

One practical inference from the findings reported here, which are consistent with parallel work on human odortypes [32], is that it should be possible to develop a detector to identify individual odortypes that can ignore environmental perturbations such as diet variation. Presuming that odor signals of individuality evolved to provide a truthful signal, which is consistent with the results reported here, individual odortypes should provide a robust alternative method to identify individuals.

## Materials and Methods

### 

#### Mice

The mice were cared for in accordance with the Guide for the Care and Use of Laboratory Animals and the experimental protocols were previously approved by the Institutional Animal Care and Use Committee in Monell Chemical Senses Center (Approval number: 900 p).

Urine donor mice and sensor mice trained in the Y maze were of the inbred strains C57BL/6J (B6: MHC type H2^b^) and its congenic partner strain C57BL/6J-H2^k^ (B6-H2^k^: MHC type H2^k^). This pair of congenic strains is genetically identical except for the small chromosomal segment containing the MHC region. All mice used in these experiments were maintained in the same animal room on a 12∶12 h light∶dark cycle.

Urine donors were all males from 20 B6 and 20 B6-H2^k^ mice. B6 mice were purchased from the Jackson Laboratory (Bar Harbor, ME). B6-H2^K^ mice were born in our laboratory. Sixteen sensor mice were used for the behavior experiments, 5 in Experiment 1 and 11 in Experiment 2. Of these, seven were B6-H2^k^ and nine were B6. No differences in the trained responses of these two MHC types were observed as has been consistently found in our studies [20].

#### Diets

All donor mice were initially fed laboratory rodent diet 5001 as purchased from Purina Mills (Diet L). Then, the 40 congenic mice (20 B6 mice and 20 B6-H2^k^) were divided into two equal groups of 20 mice/group each containing 10 B6 mice and 10 B6-H2^k^ mice. One group of 20 mice continued to be fed the same diet whereas the other group was fed a semi-synthetic diet (5755: also purchased from Purina Mills, Diet S). This resulted in four groups of 10 donor mice each with a different combination of diet (L or S) and MHC type (B6 or B6-H2^k^). Diet L contained 23.4% crude protein and 4.5% fat. Its major ingredients were: corn, soybean meal, beet pulp, fish meal, oat, yeast, molasses, alfalfa meal, whey, wheat, porcine meat meal, animal fat preserved with BHT, minerals and vitamins. Diet S, a purified, semi-synthetic diet, had 19% protein and 10% fat. Its major ingredients were: dextrin, casein, sucrose, corn oil, lard, cellulose, minerals and vitamins. All trained odor sensor mice were maintained on Diet L.

The body weight for each donor mouse was recorded 50 days after the diet change. The different diets did not significantly affect body weight in the urine donors. B6 mice fed Diet L weighed 25.8±1.72 g, B6 mice fed Diet S weighed 26.3±2.47 g, B6-H2^K^ mice fed Diet L weighed 24.62±3.42 g and B6-H2^K^ mice fed Diet S weighed 26.35±2.25 g.

#### Urine collection

Urine donors were from 10 B6 mice fed Diet L, 10 B6 mice fed Diet S, 10 B6-H2^k^ mice fed Diet L and 10 B6-H2^k^ mice fed Diet S. However, a total of 37 mice were used for the urine collection because a B6 mouse fed Diet L and 2 B6-H2^k^ mice fed Diet L died prior to the end of the experiment. Urine samples were collected individually from each mouse beginning 40 days after the diet change and continuing up to 120 days following the diet change. Voided mouse urine obtained by gentle abdominal pressure was collected directly into a sterile tube. After each collection, urine samples were frozen at −20 C until needed. For the behavioral testing, pairs of samples (each 0.3–0.4 ml) were defrosted and placed in two 3.5-cm-diameter Petri dishes.

#### Training mice in the Y-maze

The design and operation of the Y-maze apparatus used in studying odortypes are detailed elsewhere [33]. Briefly, the two arms of the maze were scented by air currents conducted through chambers containing urine in Petri dishes. For training and testing in the Y-maze, gates were raised and lowered in a timed sequence of up to 48 consecutive trials, paired urine samples being changed for each trial. During the training session, water-deprived sensor mice were rewarded with a drop of water for each correct response. After successful training (>80% concordance), unrewarded trials were interspersed, at an average frequency of one in four, with rewarded trials to accustom the mice to occasional absence of reward after a correct response. The mice performed with comparable accuracy during these trials. Mice were then tested in “generalization trials” with novel urine samples that were collected from mice with different diets and/or MHC types. This generalization procedure lends itself to blind testing of coded samples, because the operator of the maze is not required to supply reward for correct choices. Each day's training and testing in the Y-maze employed freshly-thawed urine samples maintained at room temperature.

### Behavior

#### Experiment 1

This experiment was designed to investigate how mice that were trained to discriminate urines from mice that differed both in diet and MHC type generalized this training to choices between pairs of donors that differed only either in diet or in MHC type. Five adult female mice were trained. One B6 female and one B6-H2^k^ female were rewarded in training for selecting the odor of B6 urine, fed on Diet S as opposed to B6-H2^k^ urine fed on Diet L. Two B6 females and one B6-H2^k^ female were rewarded in training for the alternative selection, B6-H2^k^ urine, fed on Diet S as opposed to B6 urine fed on Diet L. Generalization trials were then instituted so as to test four pairs of choices wherein either only diet varied with MHC type held constant or MHC type varied while diet was held constant. Specifically, the pairs of choices were: 1) urine collected from B6-H2^b^ males fed on Diet L compared with urine collected from B6-H2^k^ males fed on Diet L (bL vs. kL); 2) B6-H2^b^ males fed on Diet S from B6-H2^k^ males fed on Diet S (bS vs. kS); 3) B6-H2^b^ males fed on Diet L from B6-H2^b^ males fed on Diet S (bL vs. bS); and 4) B6-H2^k^ males fed on Diet L from B6-H2^k^ males fed on Diet S (kL vs. kS).

#### Experiment 2

The results of Experiment 1 provided no evidence that MHC type was detected when diet was varied. However, merely because the mice did not show behavioral recognition of MHC changes in the face of variation in diet does not mean that such cues were not there or that they might not be responded to in other circumstances. Thus the second behavioral experiment was designed to approach this issue differently. Here mice were trained to discriminate MHC congenic mice on one diet and their response to the same MHC difference in mice on a novel diet was tested. Three B6 males and two B6-H2^k^ females were rewarded in training for selecting the odor of B6 urine, as opposed to B6-H2^k^ urine fed on Diet L. Four B6 mice (three males and one female) and two B6-H2^k^ females were rewarded in training for the alternative selection, B6-H2^k^ urine as opposed to B6 urine fed on Diet L.

#### Chemistry

The 37 urine samples collected from each individual mouse were analyzed over a period of four days. The same SPME fiber and GC/MS were used for all analyses. The run order of the samples was randomized to minimize the analytical variability such as day to day instrumental drift, SPME fiber degradation, or ambient background differences and to ensure that comparisons between four groups were unbiased. Preliminary study also showed that there was no residual or solute cross-contamination effect between successive runs.

The method used to collect mouse urine VOCs by SPME and the parameters for the GC/MS have been described in detail elsewhere [6]. Briefly, two hundred microliters of mouse urine was placed in a 4-ml glass vial and the VOCs in the headspace sampled using a 2-cm, three-component SPME fiber (30 µm carboxen, 50 µm divinyl benzene, polydimethyl siloxane, Supelco Corp, Bellefonte, PA) at 37°C for 30 min. The SPME fiber containing the urinary VOCs was then inserted into the injection port of a Thermo-Finnigan Trace GC/MS (Thermo Electron, San Jose, CA) and desorbed for 5 min at 230°C. The GC/MS was equipped with a Stabilwax column (30 M×0.32 mm with 1.0 µ coating; Restek, Bellefonte, PA). Compound identification was accomplished through manual interpretation of mass spectra as well as matching unknowns against the NIST '02 library and comparison with commercially available standard samples when available. In addition, gas chromatographic relative retention times of all commercially available standards and mouse urine compounds were calculated relative to a series of fatty acid ethyl esters [34]. The compounds which were not commercially available, such as 2-sec-butyl-4,5-dihydrothiazole, 2-isopropyl-4,5-dihydrothiazole and 2,3-dehydro-exo-brevicomin, were tentatively identified by comparison of their mass spectra to spectra in the NIST02 mass spectral library and in the published literature [35]–[37].

#### Data analysis

Raw data files from the GC/MS system were initially processed in Matlab (version 7.0.1, The Mathworks, Inc., Natick, Massachusetts) to detect chemical components and to quantify their relative peak areas, following the general approach outlined in Willse et al. [20]. Briefly, components were detected on the basis of concomitantly peaking single ion chromatograms (SICs), because apparent peaks in TICs generally conceal multiple distinct compounds in complex mixtures like urine. For each ion trace, peaks were determined jointly across all 37 chromatograms. First, SIC peaks were detected independently for each sample, then peak locations aggregated or combined across samples into ‘consensus’ peak locations using kernel density estimation, which allows for small variations in retention times between chromatograms. Each detected component was characterized by a set of concomitantly peaking m/z values. For quantification, only m/z values were used that are well separated from neighboring peaks in their respective ion traces. Intensity values (peak areas) for components were log-transformed and organized into an *N*×*C* table, where *N* is the number of samples and *C* is the number of detected components. Mass spectra were manually examined and compared to characteristic masses determined in pre-processing, and compounds identified where possible. All subsequent analyses were conducted using the freely available R software for statistical computing [38; version 2.0.1].

The following statistical model was fit separately for each compound to assess the effects of MHC and diet on relative compound concentration:
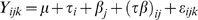
(1)where *Y_ijk_* is normalized compound concentration, *μ* is the overall average, *τ_i_* is the relative effect of MHC type *i* (i = 1,2 corresponding to B6 and B6-H2^K^), *β_j_* is the relative effect of diet *j* (*j* = 1,2 corresponding to Diet L and Diet S), and (*τβ*)*_ij_* is an interaction effect describing the extent to which the MHC effect *τ_i_* depends on diet effect *β_j_*. A significant interaction suggests that mice of different MHC types respond metabolically differently to different diets. The random error term *ε_ijk_* captures all other unexplained variation, and is assumed to have mean *0* and variance *σ* ^2^.

Although the term (*τβ*)*_ij_* in model [1] captures the interaction effect between diet and MHC, an individual interaction effect, if present, can be difficult to interpret because some interactions can be removed simply by re-scaling the data [39]. Statistical analyses in this study were performed on log-transformed peak areas, because the variance is stabilized (i.e., constant as a function of intensity) on this scale for these data. The scale at which odorants are perceived and acted on is generally not known. We therefore focus on crossover or qualitative interactions because of their unambiguous interpretation: crossover interactions cannot be removed by rescaling the data. For details of crossover interactions, see Method S1 and Figure S1.

Multivariate statistical methods were used to assess the overall salience of MHC and diet. Redundancy analysis [40] was used to decompose multivariate chromatographic profiles into their constituent sources of systematic variability corresponding to MHC, Diet, and MHC×Diet to determine which contributes most to overall variability. This is a generalization of variability decomposition performed separately for each compound, accounting for correlated (redundant) compound profiles.

Decision tree classification methodology [41] was used to construct a recursive decision tree designed to classify a sample to one of the four treatment groups based solely on its chromatogram. The decision tree produces a sequence of criteria by which a population is successively subdivided based on intensity values of certain compounds. It is expected that initial population divisions (i.e., those at the top of tree) will be based on the most salient characteristics of the population, so that in this case examination of the classification tree will provide insight into the relative salience of MHC and diet. (The term salience here refers to chemical salience not perceptual salience.) Classification trees were also used to assess how well MHC types can be classified across different diets.

To measure the importance of an individual variable (compound), Gini indexes are compared for a tree containing the variable to a tree obtained by permuting the variable. The Gini index is a measure of variability computed for each node in a decision tree, and will be 0 for a node that contains all observations assigned to same group (i.e., MHC type). For node n, let P_n1 be proportion of observations belonging to Group 1, and P_n2 be proportion belonging to group 2. Then Gini index for that node (for the 2 group case we are considering) is P_n1*(1-P_n1)+P_n2*(1-P_n2). For important variables there will be a significant increase in Gini indexes when a variable is permuted.

## Supporting Information

Method S1 Crossover interaction
(0.05 MB DOC)

Figure S1 Crossover interaction
(0.06 MB DOC)
