# Supplementary material for: Genetically-Based Olfactory Signatures Persist Despite Dietary Variation
Source: PLoS One. 2008 Oct 31;3(10):e3591. doi: 10.1371/journal.pone.0003591 (PMC2571990; doi:10.1371/journal.pone.0003591)
Supplement: Method S1 — Crossover interaction (0.05 MB DOC) [file pone.0003591.s001.doc]

Crossover interactions are illustrated in panels (a) – (c) of Figure S1. If mean group levels $\mu_{ij}=\tau_{i}+\beta_{j}+(\tau\beta)_{ij}$ from model (1) are defined as follows:

|  | **Diet L** | **Diet S** |
| --- | --- | --- |
| **B6** | $\mu_{11}$ | $\mu_{12}$ |
| **B6-H2k** | $\mu_{21}$ | $\mu_{22}$ |

then our criterion for a crossover interaction is

$sign(\mu_{11}-\mu_{12})\neq sign(\mu_{21}-\mu_{22})\text{ or }sign(\mu_{11}-\mu_{21})\neq sign(\mu_{12}-\mu_{22})$ (2).

Note that a crossover interaction is present if either of these conditions is true. Panel (a) of Figure S1 gives an example where both conditions are true. In this paper we declare a compound to have a crossover interaction effect between MHC and diet if the interaction term $(\tau\beta)_{ij}$in model (1) is statistically significant and if (2) holds, with mean values replaced by their estimates $\hat{\mu}_{ij}$.
