# Supplementary material for: Genetically-Based Olfactory Signatures Persist Despite Dietary Variation
Source: PLoS One. 2008 Oct 31;3(10):e3591. doi: 10.1371/journal.pone.0003591 (PMC2571990; doi:10.1371/journal.pone.0003591)
Supplement: Figure S1 — Crossover interaction (0.06 MB DOC) [file pone.0003591.s002.doc]

**Figure S1. Hypothetical crossover or qualitative interactions (a-c) and non-crossover or quantitative interaction (d) between MHC and diet.** The crossover interactions are characterized by reversals of mean differences. In (a),$sign(\mu_{11}-\mu_{12})\neq sign(\mu_{21}-\mu_{22})\text{ }$ and $sign(\mu_{11}-\mu_{21})\neq sign(\mu_{12}-\mu_{22})$; in (b) $sign(\mu_{11}-\mu_{12})\neq sign(\mu_{21}-\mu_{22})\text{ }$; and in (c) $sign(\mu_{11}-\mu_{21})\neq sign(\mu_{12}-\mu_{22})$. In (d) the MHC effect is greater for Diet S than for Diet L, but on a log10 scale the MHC effect is the same for both diets.

**
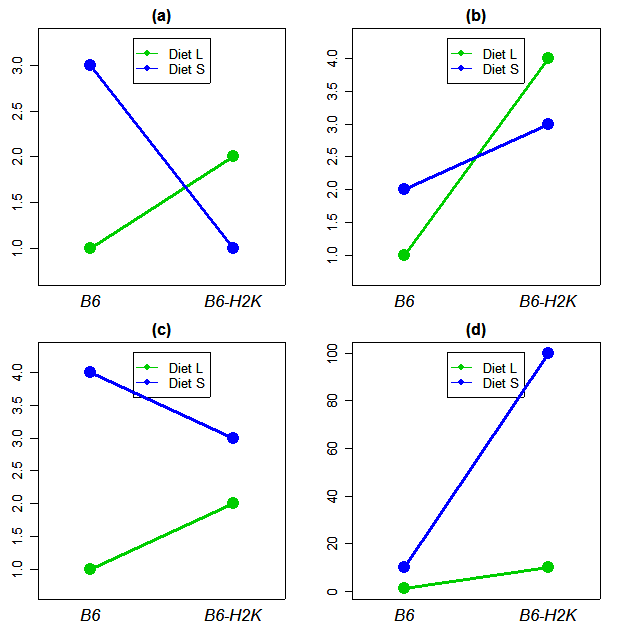
**
